# Acceptability of an automated language‐based cognitive assessment in participants with Mild Cognitive Impairment

**DOI:** 10.1002/alz70858_103305

**Published:** 2025-12-25

**Authors:** Dorota Anna Braun, Caitlin H Illingworth, Alys Griffiths, Daniel J. Blackburn

**Affiliations:** ^1^ University of Sheffield, Sheffield, South Yorkshire, United Kingdom

## Abstract

**Background:**

CognoSpeak is a fully automated language‐based assessment of cognition, developed for the stratification of patients with cognitive impairments. In light of increasing pressure on memory clinics, this tool could help patients and clinicians with screening, symptom monitoring and waitlist management. As patients with Mild Cognitive Impairment are at an increased risk of dementia many may wish to monitor their cognitive symptoms. CognoSpeak provides a potential alternative to waiting for clinically administered assessments at check‐up appointments. However, as CognoSpeak is still in development, we sought MCI patient's feedback on the best potential method of implementing this technology in the most appropriate way to benefit them.

**Methods:**

This study aims to investigate the acceptability of CognoSpeak, its potential role in the memory assessment pathway, and general attitudes toward AI and technology in healthcare among this patient group through online semi‐structured interviews and two surveys. Ten participants who have previously used CognoSpeak and identify as having mild cognitive impairment will be recruited.

**Results:**

Participants’ views on CognoSpeak, the best way to present its results, and its integration into the memory assessment pathway will be explored through semi‐structured interviews and thematic analysis. Additionally, general opinions on technology and AI will be gathered through the interviews and two structured questionnaires, The General Attitudes towards Artificial Intelligence Scale and CognoSpeak Attitude Scale, a scale created for a similar study on a healthy population.

**Conclusions:**

The results of the study will be available by June, and they will contribute to the understanding of the needs and views of patients with mild cognitive impairment on artificial intelligence and technology in healthcare.

